# Socioecological determinants of chronic disease multimorbidity in high-altitude Tibetan populations: a cross-sectional study

**DOI:** 10.1186/s41043-026-01358-y

**Published:** 2026-06-18

**Authors:** Yuanwu Zou, Lin Nan, Chen Chen, Xiaoxing Liu, Ciren Lamu, Huilan Feng, Lingxia Zeng

**Affiliations:** 1 Department of Epidemiology and Biostatistics, School of Public Health, Xi’an Jiaotong University Health Science Center, Xi’an, 710061 Shaanxi P.R. China; 2 Department of Clinical Laboratory, Shaanxi Provincial Tuberculosis Prevention and Control Hospital, Xi’an Shaanxi, 710100 P.R. China; 3 Department of Clinical Laboratory, The People’s Hospital of Ali, Tibet Autonomous Region, Ali, 859099 P.R. China; 4 Cancer Center, The First Affiliated Hospital of Xi’an Jiaotong University, Xi’an, 710061 Shaanxi P.R. China; 5 The First Affiliated Hospital of Northwest University, Xi’an, 710043 Shaanxi P.R. China; 6 Department of Child Health Care, Xi’an People’s Hospital (Xi’an Fourth Hospital), Xi’an, 710004 Shaanxi China; 7 Center for Chronic Disease Control and Prevention, Global Health Institution, Xi’an Jiaotong University, Xi’an, 710061 Shaanxi P.R. China; 8 Key Laboratory for Disease Prevention and Control and Health Promotion of Shaanxi Province, Xi’an, 710061 Shaanxi P.R. China

**Keywords:** Dietary pattern, Chronic diseases, High-altitude populations, Tibetan diet, Factor analysis

## Abstract

**Background:**

Dietary patterns are closely associated with the prevention and management of chronic diseases. Although large-scale national nutrition and health surveys have been conducted in China, evidence on dietary patterns and their associations with chronic diseases remains scarce in high-altitude regions, where unique dietary habits and lifestyles are prevalent.

**Methods:**

A cross-sectional study was conducted among high-altitude residents using food frequency questionnaires (FFQs) to assess dietary intake. Principal Component Analysis(PCA) was used to extract dietary patterns, and the scores were categorized into tertiles (from low to high: T1, T2, T3). Logistic regression models were used to examine their associations with chronic diseases, smoking, alcohol use, local tea drinking, and physical exercise.

**Results:**

Five dietary patterns were identified: dry nuts and beverages, traditional Tibetan, three-high (characterized by high salt, high carbohydrate, and high fat intake), animal-predominant high protein, and high dietary fiber. Among the 606 participants, 39.9% reported having at least one chronic disease. Fully adjusted model showed that the three-high pattern (T2 vs. T1: OR = 1.81, 95% CI: 1.10–2.99; T3 vs. T1: OR = 1.90, 95% CI: 1.09–3.33), animal-predominant high protein pattern (T2 vs. T1: OR = 2.37, 95% CI: 1.42–3.94), smoking (OR = 1.88, 95% CI: 1.06–3.35), and local tea drinking (OR = 1.85, 95% CI: 1.13–3.02) were positively associated with chronic diseases. Conversely, the traditional Tibetan pattern (T2 vs. T1: OR = 0.47, 95% CI: 0.28–0.80) and physical exercise (OR = 0.62, 95% CI: 0.39–0.99) were negatively associated with chronic diseases.

**Conclusion:**

Among high-altitude populations, traditional Tibetan pattern and physical exercise appear to be protective factors against chronic diseases, while the three-high pattern, animal-predominant high protein pattern, smoking, and local tea drinking constitute potential risk factors. Modifying dietary pattern and lifestyle thus play a significant role in the prevention and management of chronic diseases in these unique environments.

**Supplementary Information:**

The online version contains supplementary material available at https://doi.org/10.1186/s41043-026-01358-y.

## Introduction

With global modernization, developing countries are facing a growing burden of chronic non-infectious degenerative diseases, such as obesity, diabetes, cardiovascular diseases, and cancer-collectively termed “non-communicable diseases (NCDs)” or “lifestyle diseases” [1]. This trend is largely attributed to profound changes in socioecological determinants particularly diet and lifestyle over recent decades [2]. The global spread of unhealthy eating habits, dietary patterns and lifestyles has been shown to induce inflammation and oxidative stress, disrupt homeostasis and impair immune responses [2], all of which are critical factors of NCDs. Developing countries must address the lack of nutrition knowledge and health education across social groups and take measures to improve socio-demographic health [3].

Chronic diseases caused by genetic, physiological, environmental, and lifestyle factors, impose a significant economic burden [4]. The prevalence of chronic diseases is expected to rise over time, driving by an aging population, urbanization, and lifestyle changes [5]. Currently, approximately two-thirds of global deaths are attributed to chronic diseases, with the majority occurring in low- and middle-income countries [6–9]. China, home to one-fifth of the global population, is actively expanding its healthcare system to reduce the burden of disease and disability [10,11]. However, achieving Sustainable Development Goal 3.4 (a 25% reduction of non-communicable diseases mortality by 2025) and Healthy China 2030 (a one-third reduction in premature mortality from non-communicable diseases by 2030) remains a significant challenge [12]. Chronic diseases reduce labor productivity, increase healthcare costs, and hinder social and economic development, becoming a critical public health concern threatening Chinese residents’ health [13,14].

Modifiable lifestyle factors such as diet, physical exercise, smoking, and alcohol consumption, play a critical role in the management of chronic diseases [15–20]. Among these, diet and lifestyle exhibit a synergistic impact on overall health. Economic globalization has diversified food supplies, prompting many developing countries to shift away from traditional dietary patterns toward Western-style eating habits [21]. Epidemiological studies on nutrition and diseases have progressively shifted its focus from a single nutrient to the examination of whole-food combinations [22–24]. Dietary pattern analysis accounts for complex interactions among dietary components that influence nutrient bioavailability [25,26]. Evaluating the cumulative effects of multiple components, rather than single nutrients, provides deeper insights into the relationship between dietary intake and chronic diseases.

Geographical location and place of residence significantly influence food choices and dietary habits [27,28]. Alpine regions are characterized by limited food variety and scarce fish consumption [29], while high-temperature regions rely heavily on staple grains, roots, and tubers for energy [30]. To promote national health initiatives, China issued the *2022 Dietary Guidelines for Chinese Residents*, introducing the “Oriental Healthy Dietary Pattern”. This pattern, prevalent in southeast coastal areas, is characterized by a high intake of vegetables, fruits, aquatic products, soy products, and milk, along with low consumption of salt and light cooking, which significantly reduced hypertension and cardiovascular disease incidence and mortality in these regions [31]. Despite extensive national surveys in China, specific studies on high altitude areas such as Tibet with distinct dietary preferences remain scarce. The Ali Region (northern Qinghai-Tibet Plateau, average altitude > 4,500 m) has unique geography and climate, leading to a grazing lifestyle with low intake of vegetables, fruits, seafood and grains, and high red meat, milk and animal fat consumption [32]. However, the association between its dietary patterns and chronic diseases remains unclear.

Given above, the primary aim of this study is to identify dietary patterns in high-altitude areas and investigate their relationship with chronic diseases, and the findings are intended to provide evidence for lifestyle intervention strategies to manage these diseases.

## Methods

### Study design, setting and participants

**Fig. 1:**
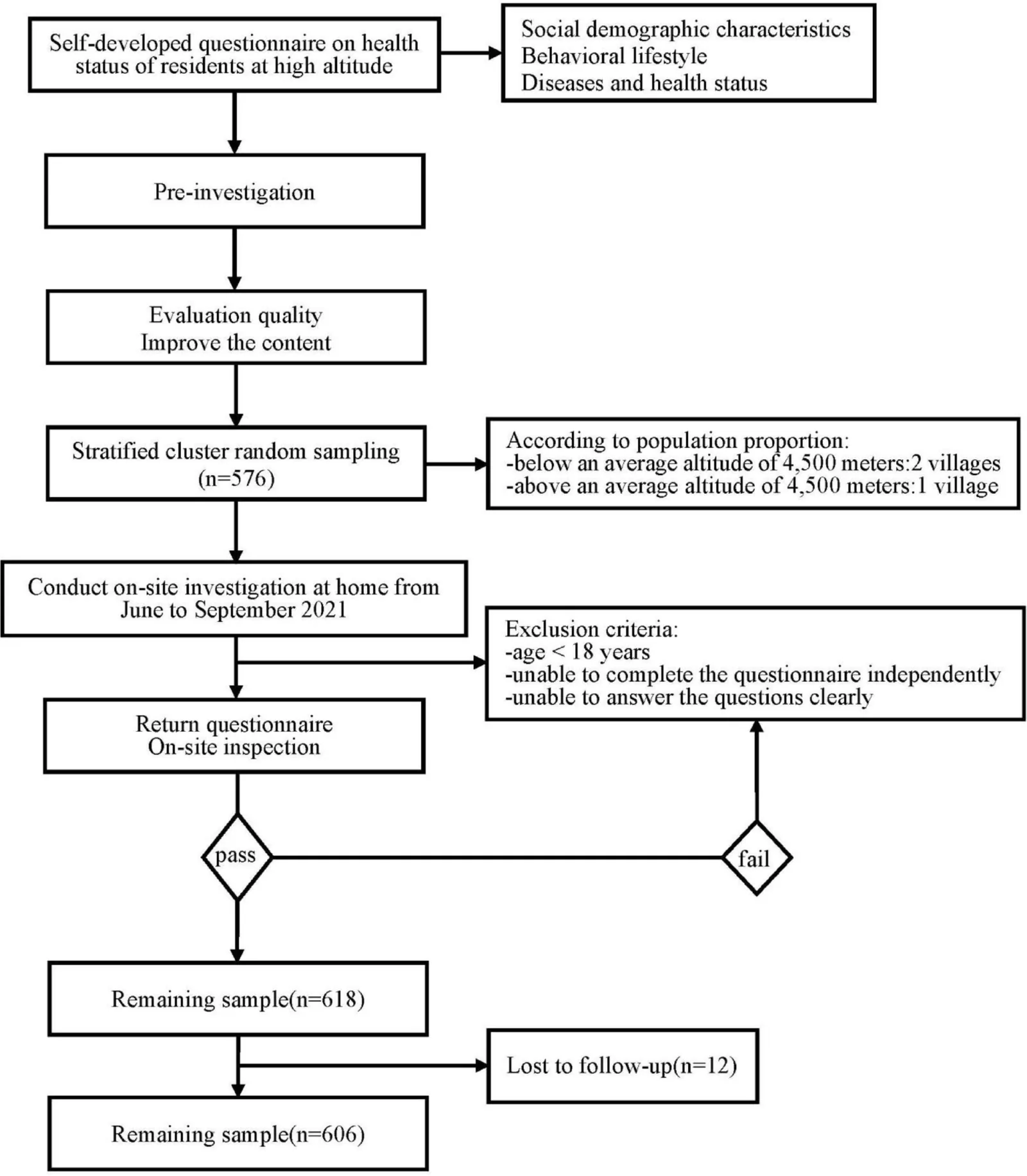
Flow chart for investigation

A cross-sectional study employing stratified cluster random sampling was conducted in Ali, Tibet Autonomous Region, between June and September 2021. Based on altitude, the seven counties were classified into two categories: average altitude below 4,500 m and above 4,500 m. Three administrative villages were randomly selected for the population survey. Permanent residents aged 18 years and older in Ali were included, with the exception of individuals if they exhibited severe cognitive impairment, communication barriers, or severe mental disorders that prevented them from understanding the questions (Fig. 1). The study was approved by the Ethics Committee of Ali People’s Hospital and obtained written informed consent from all participants.

### Sample size estimation

The minimum required sample size was calculated as 315 using the formula: $n=\frac{\rm{Z}^2_{1 - \alpha / 2} \times \rm{pq}}{\delta^2}$. A crude prevalence of chronic diseases in this area at 55.0% [33], an allowable error (δ) of 5.5%, and a significance level (α) of 0.05. Accounting for 20% non-response rate and a design effect of 1.5 for cluster sampling, the final sample was size was adjusted to 567.

### Data collection

A semi-quantitative food frequency questionnaire (FFQ) was integrated into a general health questionnaire for residents living in high-altitude areas. The FFQ was developed based on previously validated FFQs used in similar high-altitude and Tibetan populations to reflect local diet culture and lifestyle [34,35], covered sociodemographic characteristics, dietary intake, behavioral lifestyles, and health status. Data were collected through face-to-face interviews conducted by well-trained investigators to ensure consistency and accuracy. A total of 618 questionnaires were distributed, of which 606 were deemed valid, resulting in a response rate of 98.1%.

### Information about chronic diseases

This study investigated twenty-two chronic diseases, including diabetes, acute myocardial infarction, angina pectoris, other ischaemic heart disease, stroke, hypertension, pulmonary heart disease, rheumatic heart disease, tuberculosis, emphysema, chronic bronchitis, chronic obstructive pulmonary disease (COPD), asthma, chronic hepatitis or cirrhosis, peptic ulcer, gallstones or cholecystitis, chronic kidney disease, osteoporosis, rheumatic arthritis, psychological disorders (depression, anxiety, neurasthenia, etc.), cancer, and other chronic diseases. Chronic diseases were self-reported by participants based on their previous clinical diagnoses and validated in the healthcare system.

### Assessment of dietary intake

A total of 47 food items with Tibetan cultural characteristics were selected in the FFQ. Respondents were asked to recall the frequency and portion size of each food item consumed over the past 12 months. Portions were measured using local weight units (1Liang=50 g). Food intake frequency was categorized into six groups: none or very rarely, 1–3 times/month, 1–3 times/week, 4–6 times/week, and daily. Prior to the formal survey, 30 individuals were randomly selected for a plot investigation. The FFQ showed high reliability with a Cronbach’s alpha coefficient of 0.943, and the Kaiser-Meyer-Olkin (KMO) test yielded a value of 0.931.

### Data associated with behavioral lifestyle

Behavioral lifestyles were evaluated based on: smoking, alcohol drinking, local tea drinking, physical exercise, and significant body weight changes in the past year. Smoking was defined as smoking either daily or most days (averaging at least one cigarette per day). Alcohol drinking was defined as any drinking behavior other than never or occasional drinking. Local tea drinking consists of fermented dark brick tea, salt combined with butter to form butter tea, and sweet tea composed of black tea salt, sugar and whole milk. It was categorized similarly to alcohol drinking. Engaging in physical activity more than once a month was considered as participation in regular physical exercise. Significant body weight changes in the past year were assessed with the question: “Did your weight change significantly in the past year?” Response options included: weight change of no more than 2.5 kg, weight gain of more than 2.5 kg, and weight loss more than 2.5 kg.

In the baseline survey, social demographic characteristics were collected, including gender, ethnicity, age, education level, marital status, employment status, household assets and facilities, and family size. Education level was categorized into primary school and below, middle school, and college or above. Marital status was classified as “in marriage (married or remarried) and non-in marriage” (unmarried, separated, divorced, or widowed). Household assets or facilities including apartments, private flush toilets, automobiles, motorcycles/other motor vehicles, computers, internet access, smartphones, and self-funded travel within the past five years. Self-rated health (SRH) was assessed through self-report using the question: “How would you rate your current health?” with options: excellent, good, fair, and poor.

### Data quality control

All investigators underwent standardized training in questionnaire management training before the survey began, ensuring at least one team member was proficient in both Tibetan and Mandarin. After completing the survey, investigators cross-checked the questionnaire. If key information was missing or variable values were abnormal, investigators conducted follow-up surveys.

### Statistical analysis

Statistical analysis was performed using Stata15.0 (Stata Corp, College Station, Texas, USA). The two-tailed test *P* value less than 0.05 was considered statistically significant. Continuous variables were presented as mean with standard error (SE). Categorical variables were presented as percentages (%) and differences between groups were examined by χ2 test. The household wealth index was constructed by principal component method (PCA) based on household assets or facilities. Based on the first principal component, it was divided into low, medium and high levels by tertile method, reflecting household wealth. Principal component analysis combined with factor analysis was employed to identify dietary patterns. Moreover, the factors were rotated using Varimax orthogonal method to enhance interpretability. Dietary patterns were characterized based on high factor loadings (absolute value ≥ 0.30) and eigenvalues ≥ 1.50, and named in accordance with the component loadings and local dietary characteristics. We divided the factor scores of each dietary pattern into T1, T2, and T3 in ascending order using the tertile method for further analysis. Correlations of dietary patterns with other factors were performed using contingency coefficient and gamma coefficient. Variance Inflation Factor (VIF) ranged between 1.05 and 3.40 for all independent variables, without significant multicollinearity. The linearity of the logit link was examined using the Box-Tidwell test, and no violations of the linearity assumption were detected. Multivariate logistic regression was used to assess the association between behavioral lifestyle, dietary patterns and chronic diseases. Model 1 (unadjusted model): dietary patterns, smoking, alcohol drinking, local tea drinking and physical exercise were included. Model 2 (basic adjusted model): adjusted for age, education level, and employment status. Model 3 (fully adjusted model): further adjusted for ethnic group, household wealth, number of family members, body weight change in the past year, and self-rated health.

## Results

### Respondent characteristics and prevalence of chronic diseases

A total of 606 respondents completed the survey, with ages ranging from 18 to 92 years and a mean age of 40.7 years (SD, 16.4). The majority were Tibetan (81.4%) (Table 1).

**Table 1:** Characteristics and prevalence of chronic diseases among participants in Ali area, 2021 (*n* = 606)

Variables	Number	Proportion (%)	Prevalence (*n*(%))	*P*-value
Total	606	100.0	242 (39.9)	
Gender				0.466
Male	329	54.3	127 (38.6)	
Female	277	45.7	115 (41.5)	
Ethnic group				0.003
Other	113	18.6	31 (27.4)	
Tibetan	493	81.4	211 (42.8)	
Age				< 0.001
≤ 29	175	28.9	40 (22.9)	
30–49	264	43.6	98 (37.1)	
≥ 50	167	27.5	104 (62.3)	
Education level				< 0.001
Primary school and below	273	45.1	151 (55.3)	
Middle school	168	27.7	49 (29.2)	
College and above	165	27.2	42 (25.5)	
Marital status				0.530
In marriage	422	69.6	172 (40.8)	
Non-in marriage	184	30.4	70 (38.0)	
Employment status				< 0.001
Administration and management	126	20.8	48 (38.1)	
Professional technicians	140	23.1	31 (22.1)	
Housework	156	25.7	68 (43.6)	
Other	184	30.4	95 (51.6)	
Household wealth				0.001
Low	207	34.2	98 (47.3)	
Medium	214	35.3	91 (42.5)	
High	185	30.5	53 (28.6)	
Number of family members*				0.350
0–3	309	51.5	118 (38.2)	
≥ 4	291	48.5	122 (41.9)	
Smoking				0.831
No	511	84.3	205 (40.1)	
Yes	95	15.7	37 (38.9)	
Alcohol drinking				0.989
No	521	86.0	208 (39.9)	
Yes	85	14.0	34 (40.0)	
Local tea drinking				0.005
No	179	29.5	56 (31.3)	
Yes	427	70.5	186 (43.6)	
Physical exercise				< 0.001
No	393	64.9	181 (46.1)	
Yes	213	35.1	61 (28.6)	
Body weight change in the past year				0.385
Weight change < 2.5 kg	446	73.6	172 (38.6)	
Weight gain ≥ 2.5 kg	86	14.2	40 (46.5)	
Weight loss ≥ 2.5 kg	74	12.2	30 (40.5)	
Self-rated health				< 0.001
Well	354	58.4	92 (26.0)	
General	166	27.4	84 (50.6)	
Poor	86	14.2	66 (76.7)	

Prevalence was the prevalence of one or more chronic diseases. * six participants were missing data for number of family members

**Fig. 2:**
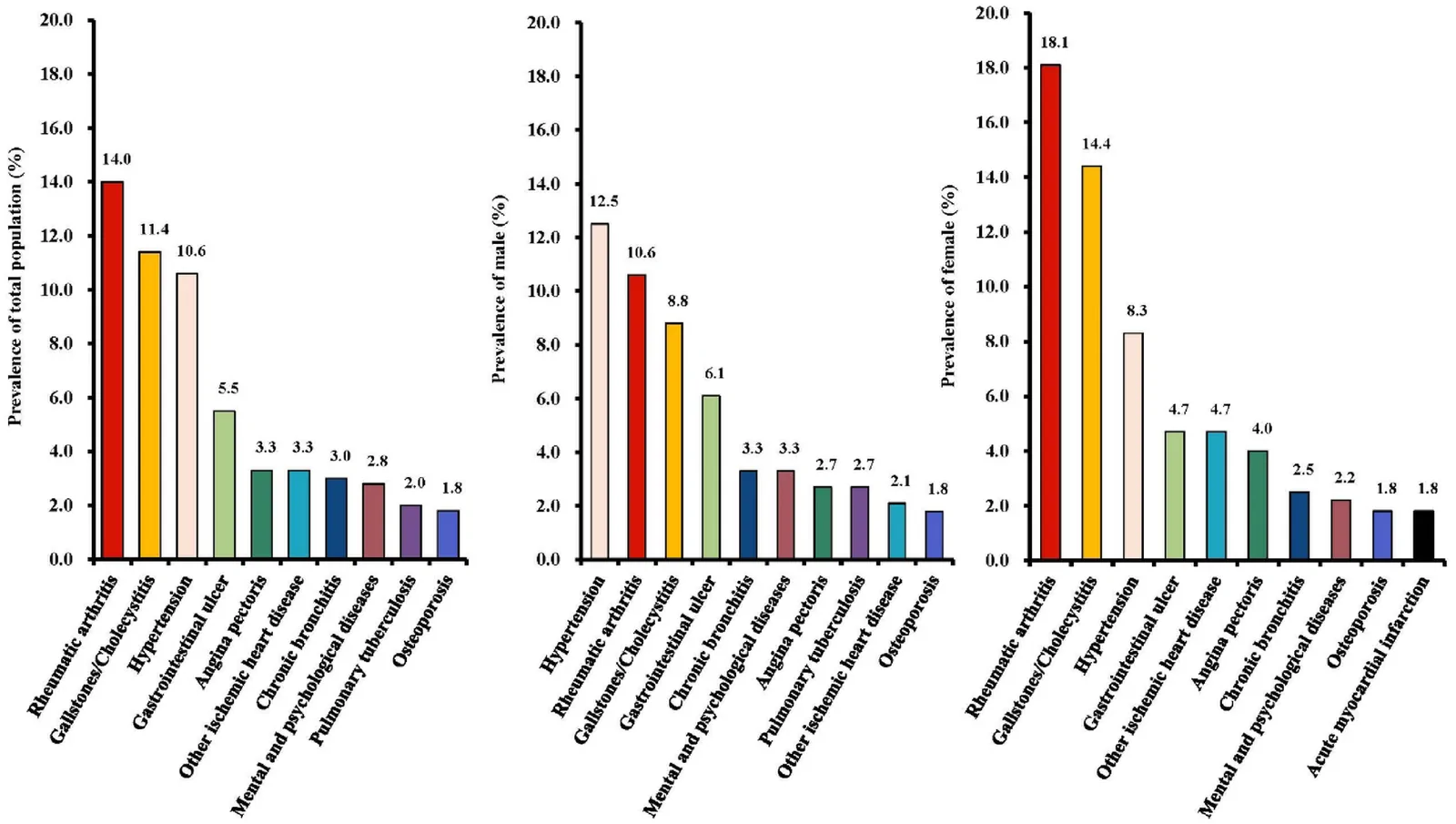
The top 10 chronic diseases in Ali area, 2021

**Fig. 3:**
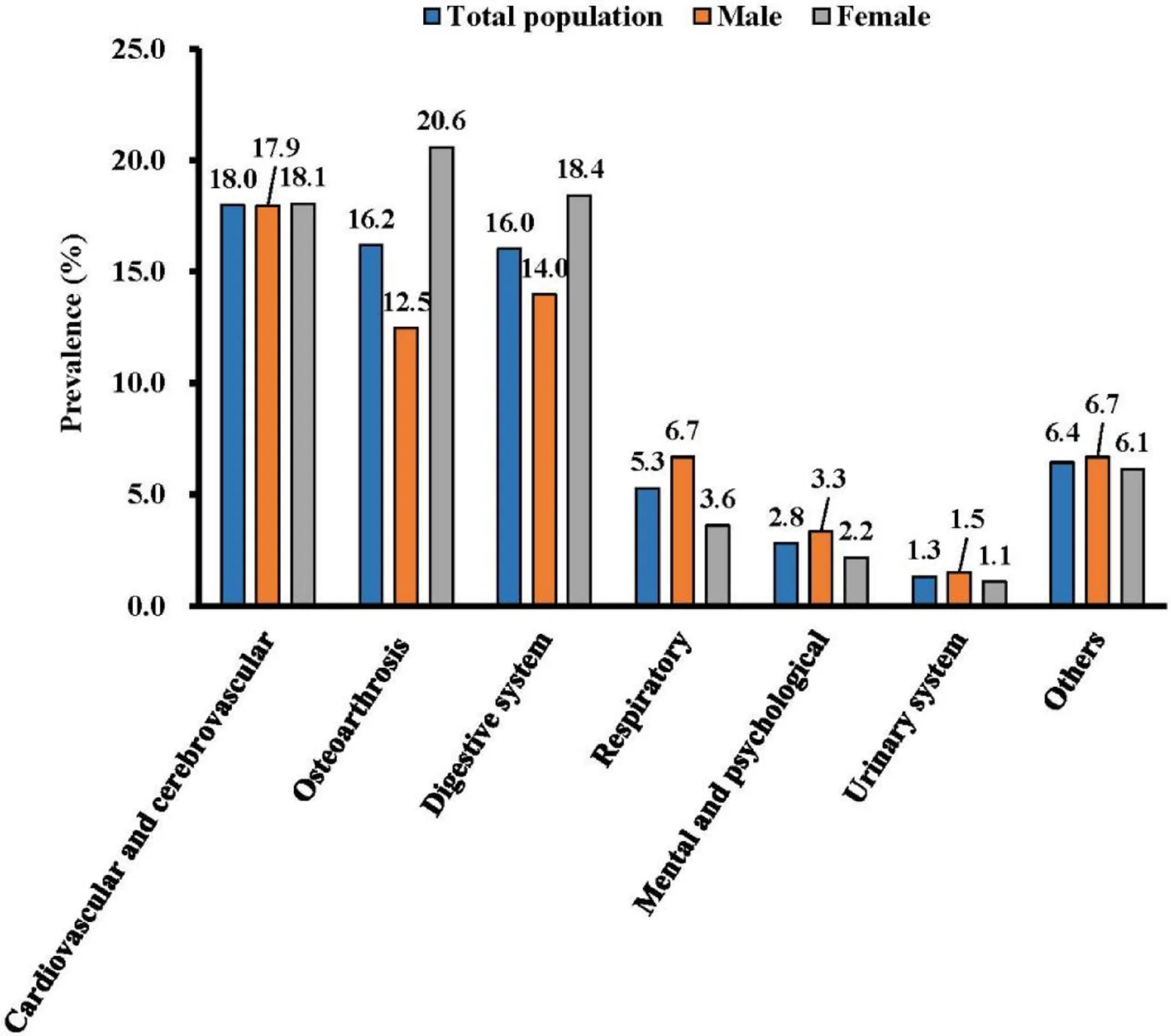
Diseases of sick organ system in Ali area, 2021

A total of 242 residents reported having at least one chronic disease. The ranking of the ten most prevalent chronic diseases differed between males and females (Fig. 2). Among both the overall population and males, cardiovascular and cerebrovascular diseases ranked first, while osteoarthrosis was the most prevalent among females (Fig. 3).

### Associations between demographic characteristics, health status and chronic diseases

**Fig. 4:**
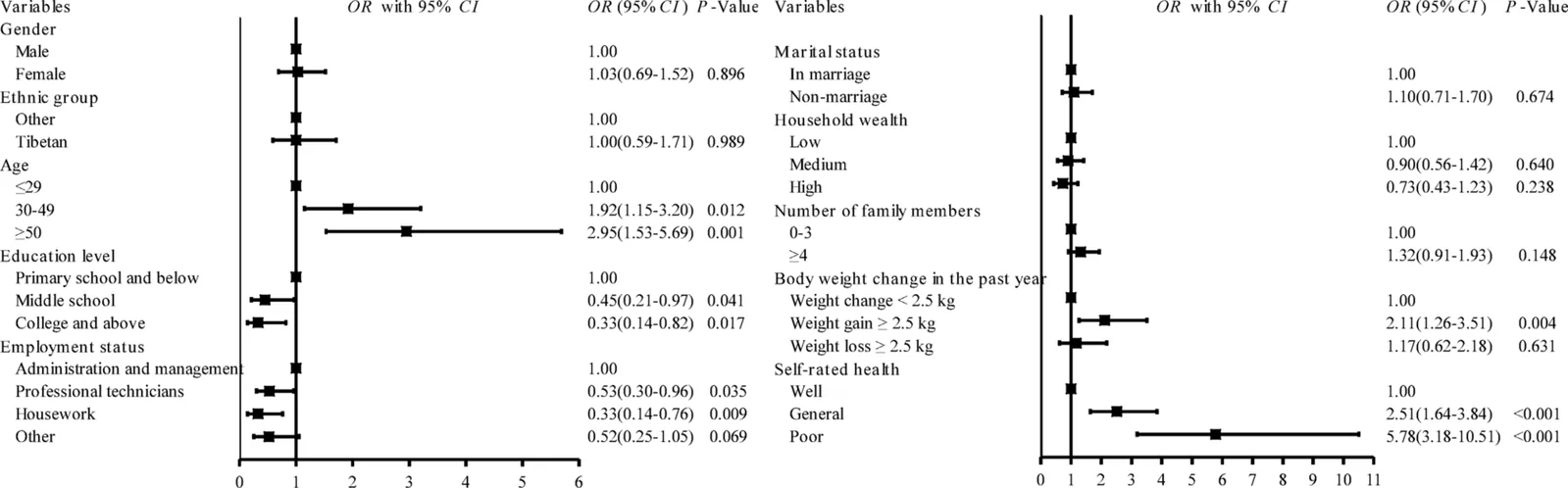
Association between participant sociodemographic characteristics and chronic diseases

This study identified statistical differences in chronic disease prevalence among ethnic groups, age categories, educational levels, employment statuses, household wealth, and self-rated health (Table 1). Age, weight gain, and poorer self-rated health were positively associated with chronic diseases. In contrast, higher educational levels and certain employment status (professional technicians vs. administrative roles: OR = 0.53, 95% CI:0.30–0.96; housework vs. administrative roles: OR = 0.33, 95% CI:0.14–0.76) exhibited negative associations with chronic diseases.(Fig. 4).

### Characteristics of dietary patterns

**Fig. 5:**
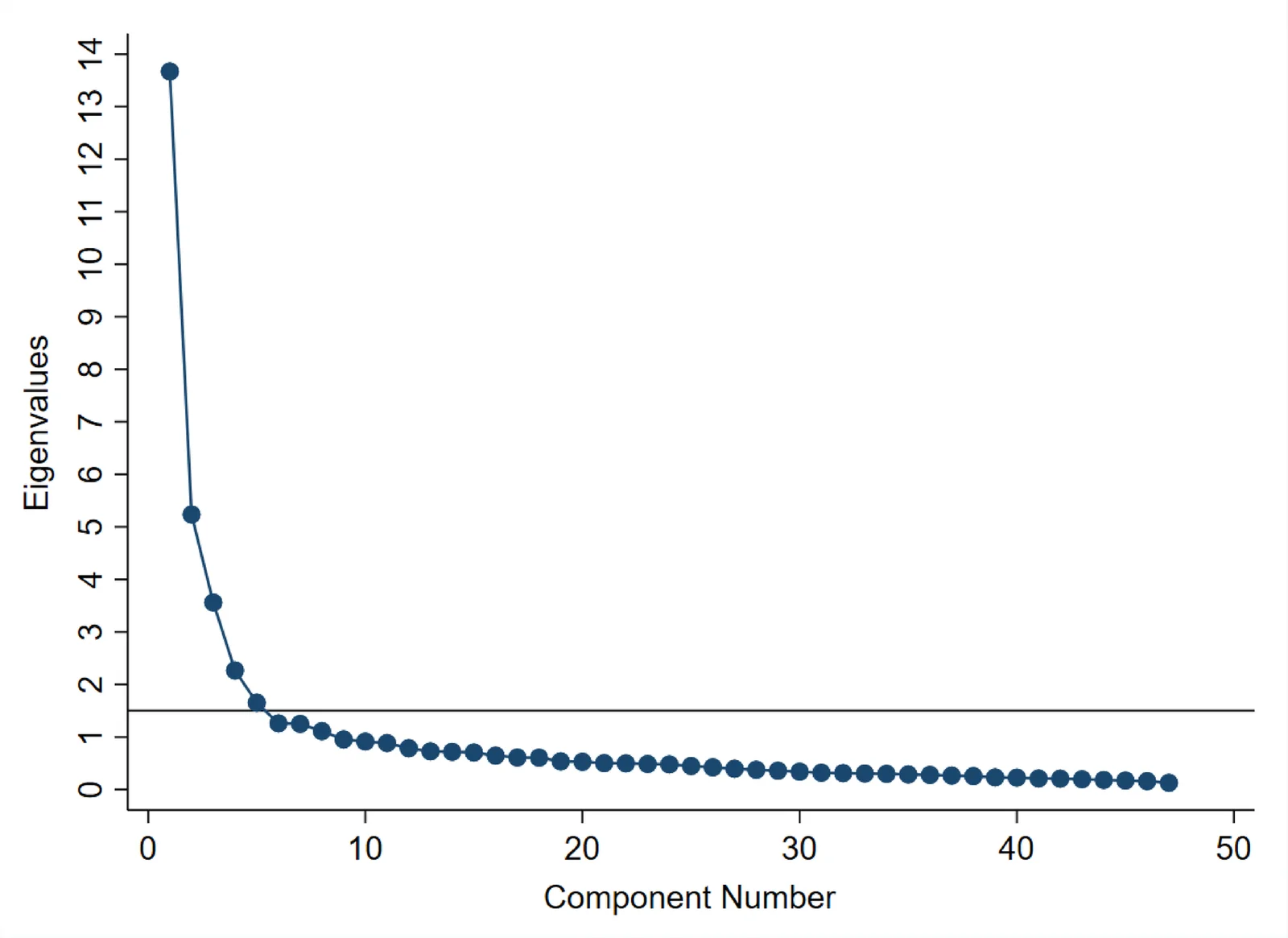
Scree plot of factor analysis

Five main dietary patterns were identified, explaining 53.16% of the total variance (Fig. 5). The KMO measure of sampling adequacy was 0.931, and Bartlett’s test of sphericity was significant (*χ*^2^ = 17753.44, *P* < 0.001). These dietary patterns were characterized as follows: Factor 1: Dry nuts and beverage pattern-Includes salted pickles, various dried fruits, and carbonated drinks; Factor 2: Traditional Tibetan pattern-Features high-energy foods, such as beef, mutton, dairy products, butter, tsampa, butter tea and dried meat; Factor 3: Three-high pattern-Comprise snacks, convenience foods, midnight snacks, bacon, processed meat, barbecue, fried foods, western fast food, eating out and Tibetan brown sugar; Factor 4: Animal-predominant high protein pattern-Key components include pork, poultry, aquatic products/seafood and animal viscera; Factor 5: High dietary fiber pattern-Mainly include vegetables, fruits, potatoes and beans) (Table 2).

**Table 2:** Factor loadings and explained variation of dietary patterns from PCA

Type of food	Dry nuts and beverage	Traditional Tibetan	Three-high	Animal-predominant high protein	High dietary fiber
Salted pickles	**0.619**				
Dried vegetable	**0.786**				
Nuts	**0.858**				
Jujube	**0.847**				
Wolfberry	**0.816**				
Raisins	**0.828**				
Other dried fruit	**0.863**				
Soy milk	**0.673**				
Fruit and vegetable juice	**0.673**				
Carbonated beverages	**0.460**				
Other drinks	**0.661**				
Tsampa		**0.797**			
Butter		**0.790**			
Pasta		**0.436**			
Mutton		**0.773**			
Beef		**0.649**			
milk		**0.758**			
Yogurt		**0.697**			
Other dairy products		**0.532**			
Butter tea		**0.768**			
Milk tea		**0.330**			
Air-dried meat		**0.648**			
Cheese		**0.563**			
Snacks			**0.721**		
Convenience food			**0.766**		
Midnight snack			**0.507**		
Bacon			**0.612**		
Processed meat			**0.783**		
Without breakfast			**0.430**		
Fried food			**0.670**		
Barbecue			**0.766**		
Western fast food			**0.611**		
Eating out			**0.642**		
Tibetan brown sugar			**0.447**		
Miscellaneous grain				**0.322**	
Pork				**0.511**	
Poultry and products				**0.610**	
Aquatic products/Seafood				**0.693**	
Eggs				**0.542**	
Animal viscera				**0.632**	
Rice					**0.331**
Vegetables					**0.691**
Fruit					**0.744**
Potatoes					**0.689**
Beans					**0.470**
Variance Explained (%)	17.13	12.03	11.96	6.31	5.73

* the five groups with highest factor loadings in each dietary pattern were presented in bold

The correlation analysis of dietary patterns revealed that the dry nuts and beverage pattern was significantly correlated with marital status, employment status, and physical exercise. The traditional Tibetan pattern showed strong correlations with gender, ethnic group, age, education level, employment status, household wealth, number of family members, smoking, alcohol drinking, physical exercise, and body weight change over the past years. The three-high pattern was correlated with gender, age, marital status, employment status, smoking, local tea drinking, physical exercise, and body weight change over the past year. The animal-predominant high protein pattern was associated with gender, ethnic group, employment status, smoking, alcohol drinking, local tea drinking, and physical exercise. The high dietary fiber protein pattern presented significant associations with gender, ethnic group, employment status, local tea drinking, and physical exercise (Table 3).

**Table 3:** Correlation analysis between dietary pattern and other variables

Variables	Dry nuts and beverage	Traditional Tibetan	Three-high	Animal-predominant high protein	High dietary fiber
Gender	0.05	0.15^**^	0.11^*^	0.13^**^	0.10^*^
Ethnic group	0.06	0.51^***^	0.06	0.11^*^	0.14^**^
Age	0.04	-0.37^*^	0.47^*^	0.13	0.04
Education level	-0.24	0.73^*^	-0.30	-0.15	-0.19
Marital status	0.10^*^	0.05	0.12^**^	0.08	0.01
Employment status	0.23^***^	0.48^***^	0.21^***^	0.17^**^	0.17^**^
Household wealth	-0.10	0.45^*^	-0.26	-0.16	-0.24
Number of family members	0.01	0.14^**^	0.02	0.03	0.02
Smoking	0.02	0.26^***^	0.18^***^	0.11^*^	0.09
Alcohol drinking	0.04	0.17^***^	0.02	0.16^***^	0.06
Local tea drinking	0.02	0.10	0.11^*^	0.11^*^	0.12^*^
Physical exercise	0.15^**^	0.24^***^	0.10^*^	0.12^*^	0.12^*^
Body weight change in the past year	0.10	0.22^***^	0.19^***^	0.08	0.11
Self-rated health	0.38	-0.32	0.22	0.21	0.14

Unidirectional ordered variables used contingency coefficients; Bidirectional ordered variables used gamma coefficients. *P < 0.05, **P < 0.01, ***P < 0.001

### Associations between dietary patterns, behavioral lifestyle and chronic diseases

Binary logistic regression was used to assess the associations between dietary patterns, behavioral lifestyle, and chronic diseases in both unadjusted and adjusted models (Table 4). Model 1 (unadjusted variables) showed positive associations of higher scores in three-high pattern, the animal-predominant high protein pattern, and local tea drinking with chronic diseases, while the traditional Tibetan pattern and participation in physical exercise showed negative associations with such conditions. In Model 2, after adjusting for age, educational level and employment status, the three-high pattern (T2:*OR* = 1.68, 95% *CI*:1.04–2.71; T3:*OR* = 1.82, 95% *CI*:1.08–3.07), middle tertile of the animal-predominant high protein pattern (T2:*OR* = 2.44, 95% *CI*:1.50–3.98), smoking (*OR* = 1.79, 95% *CI*:1.02–3.14) and local tea drinking (*OR* = 2.55, 95% *CI*:1.63–4.00) were positively associated with chronic diseases compared with the reference group. The middle tertile of the traditional Tibetan pattern (*OR* = 0.48, 95% *CI*:0.30–0.77) and participation in physical exercise (*OR* = 0.59, 95% *CI*:0.38–0.91) were negatively associated with chronic diseases. In Model 3, after further adjusting for ethnicity, household wealth, number of family members, body weight change in the past year, and self-rated health, the aforementioned associations remained unchanged.

**Table 4:** Multivariate logistic regression analysis of the association between dietary patterns, behavioral lifestyle and chronic diseases

Variables	Model 1		Model 2		Model 3	
	OR(95%CI)	*P*-value	OR(95%CI)	*P*-value	OR(95%CI)	*P*-value
Dry nuts and beverage						
T1	1.00		1.00		1.00	
T2	0.81(0.51–1.30)	0.385	0.76(0.47–1.23)	0.257	0.63(0.37–1.07)	0.085
T3	1.46(0.92–2.32)	0.112	1.30(0.80–2.11)	0.295	0.98(0.59–1.64)	0.952
Traditional Tibetan						
T1	1.00		1.00		1.00	
T2	0.39(0.25–0.60)	< 0.001	0.48(0.30–0.77)	0.003	0.47(0.28–0.80)	0.006
T3	0.43(0.26–0.69)	0.001	0.84(0.46–1.52)	0.564	0.94(0.48–1.84)	0.853
Three-high						
T1	1.00		1.00		1.00	
T2	2.04(1.29–3.23)	0.002	1.68(1.04–2.71)	0.033	1.81(1.10–2.99)	0.020
T3	3.17(1.95–5.17)	< 0.001	1.82(1.08–3.07)	0.024	1.90(1.09–3.33)	0.024
Animal-predominant high protein						
T1	1.00		1.00		1.00	
T2	2.41(1.52–3.80)	< 0.001	2.44(1.50–3.98)	< 0.001	2.37(1.42–3.94)	0.001
T3	1.71(1.08–2.73)	0.023	1.47(0.91–2.38)	0.118	1.21(0.72–2.01)	0.472
High dietary fiber						
T1	1.00		1.00		1.00	
T2	1.08(0.68–1.72)	0.749	0.99(0.62–1.60)	0.977	0.97(0.59–1.61)	0.908
T3	0.92(0.58–1.46)	0.715	0.83(0.51–1.35)	0.451	0.62(0.36–1.05)	0.076
Smoking						
No	1.00		1.00		1.00	
Yes	1.61(0.91–2.84)	0.099	1.79(1.02–3.14)	0.042	1.88(1.06–3.35)	0.031
Alcohol drinking						
No	1.00		1.00		1.00	
Yes	1.12(0.64–1.94)	0.691	1.19(0.67–2.13)	0.552	1.24(0.68–2.25)	0.478
Local tea drinking						
No	1.00		1.00		1.00	
Yes	2.23(1.48–3.36)	< 0.001	2.55(1.63-4.00)	< 0.001	1.85(1.13–3.02)	0.015
Physical exercise						
No	1.00		1.00		1.00	
Yes	0.49(0.33–0.74)	0.001	0.59(0.38–0.91)	0.017	0.62(0.39–0.99)	0.044

T1: lowest tertile of dietary pattern scores; T2: middle tertile of dietary pattern scores; T3: highest tertile of dietary pattern scores. Model 1: unadjusted; Model 2: adjusted for age, education level, and employment status; Model 3: further adjusted for ethnic group, household wealth, number of family members, body weight change in the past year, and self-rated health

## Discussion

The global proliferation of unhealthy dietary patterns and lifestyles has been linked to elevated inflammation and oxidative stress, impaired metabolic homeostasis, and dysregulated immune function-all of which represent key pathogenic mechanisms underlying the development of non-communicable diseases (NCDs). The cuisine of the Ali region is distinctive, shaped by diverse regional cultures, traditional religious beliefs, and food taboos, and research focusing on subgroups in this region remains limited [36]. This study is one of the latest to explore the association between dietary patterns and chronic diseases at high altitude. Moreover, the findings reflect the dual impact of traditional and modernized dietary practices on chronic diseases.

The prevalence of chronic diseases was 39.9% among the surveyed population and higher among Tibetan participants. This exceeds the prevalence of 33.1% reported for residents aged over 15 years in China’s Fifth National Health Service Survey in 2013 [37]. It is encouraging that the prevalence of chronic diseases in this survey is lower than previously reported for the same region [33]. Rheumatoid arthritis ranking first among the surveyed population is likely due to the high altitude and cold climate [38].

Logistic regression analyses confirmed that older age, poorer self-rated health, and the body weight gain of over 2.5 kg in the past year were positively associated with chronic diseases. These findings align with those of previous studies [39–41] highlighted the need for targeted prevention efforts in older adults’ poor health and weight gain tendencies. Moreover, professional technicians and individuals engaged in housework showed a negative association, possibly due to reduced sedentary time compared with those in administrative or management roles [42]. Additionally, housework may contribute favorably to achieving recommended daily physical activity levels. Higher educational levels were protective against chronic diseases, possibly due to increased self-care awareness and health literacy.

Five distinct dietary patterns were identified, reflecting the unique local dietary characteristics. The dry nuts and beverage pattern encompassed a diverse array of dry nuts and beverages. The traditional Tibetan pattern was characterized by high-energy foods. The three-high pattern, characterized by high salt, sugar, and fat, represents unhealthy dietary behaviors, such as consuming processed meat. The animal-predominant high protein pattern is abundant in animal-derived proteins. A healthy dietary pattern rich in dietary fiber, primarily derived from fruits and vegetables, was also observed, which has not been reported in previous studies [32]. However, a balanced dietary pattern incorporating meat, eggs, vegetables, fruits, beans and seafood was absent [43]. With urbanization influencing traditional culture, local dietary patterns align with China’s recent nutritional transition [44]. These changes explain the emergence of unhealthy three-high and animal-predominant high protein patterns.

Dietary patterns are influenced by various factors. The dry nuts and beverage pattern, often considered unhealthy, correlates positively with employment status, reflecting cognitive differences in health literacy across occupations [45]. The traditional Tibetan pattern is positively associated with educational level, employment status, and household wealth, but negatively correlated with age. This relatively expensive dietary pattern is well suited to the local environment. Well-educated individuals may select foods aligned with their economic status, and higher income supports such consumption. Younger generations, influenced by global economic and culture, tended towards western dietary preferences [46]. In contrast, older residents are more likely to follow the three-high pattern due to lower educational attainment and economic development in less developed areas, contributing to reduced health literacy [47]. The animal-predominant high protein pattern and high dietary fiber pattern showed weaker associations with these factors.

The three-high pattern and animal-predominant high protein pattern were identified as significant risk factors for chronic diseases, while the traditional Tibetan pattern emerged as a protective factor. However, the mechanisms underlying these associations remain unclear. Evidence from previous studies suggests that the diet high in salt, fat and sugar [48–51], as exemplified by the three-high pattern, particularly due to processed foods-may increase the risk of chronic diseases through metabolic disruptions [52]. Similarly, diets rich in animal protein, dominated by red meat due to the limited availability of local aquatic products, may contribute to the progression of chronic diseases (i.e., chronic kidney diseases) [53,54].

The traditional Tibetan pattern is a distinctive dietary pattern developed by local residents to adapt to the geographical environment and cultural traditions, which is characterized by large consumption of red meat, dairy products, butter tea and tsampa. While research has indicated that excessive consumption of red meat (high in saturated fat and cholesterol) can lead to excessive energy intake and an increased risk of chronic diseases [55], our study hypothesized that the traditional Tibetan pattern may confer protective effects against chronic diseases in the context of the plateau environment. The unique environmental conditions may necessitate a higher energy intake, which this dietary pattern appears to fulfills. Additionally, tsampa-a staple food made from roasted highland barley flour-is rich in dietary fiber. The total dietary fiber content of highland barley flour is significantly higher than that of barley, wheat, oat and other cereal crops, making it a potent protective factor against chronic diseases.

]Moreover, plant-based foods are widely recognized to benefit chronic diseases management [56–59] owing to their abundant content of antioxidants and anti-inflammatory compounds, including vitamin C, β-carotene and dietary fiber [60,61]. However, we did not observe a statistically significant protective effect of the high dietary fiber pattern against chronic diseases. This may be attributable to insufficient intake of plant-based foods or the possibility that the dietary pattern primarily affects specific types of chronic diseases. Furthermore, the availability and diversity of local vegetables and fruits are limited by the plateau environment and climate, which hinder plant growth and, consequently, the consumption of such foods.

Unhealthy behavioral lifestyles, such as physical inactivity, smoking and alcohol abuse, contribute to metabolic alterations and represent well-established risk factors for chronic diseases [4]. Consistent with this understanding, our findings indicate that smoking and local tea consumption are positively associated with chronic diseases while physical exercise serves as a protective factor. The beneficial health effects of tea consumption on chronic diseases have been widely reported [62–64], with phytochemicals in tea regulating metabolism primarily through antioxidant effects [65]. However, influenced by local traditional food culture, the tea composition in this paper differs from previous studies, mainly butter tea and sweet tea. Butter tea is made by combining fermented dark brick tea salt, and butter, while sweet tea consists of black tea, salt, sugar and whole milk. Both types of tea can lead to excessive lipid accumulation, contributing to a higher risk of metabolic disorders [49,51]. Additionally, our study confirmed that smoking and physical exercise-modifiable behavioral factors [39]-play a crucial role in the development of chronic diseases.

### Strengths and limitations

To our knowledge, this is the most recent study to explore the associations between dietary patterns and chronic diseases among residents of high-altitude regions. Using survey tools designed to reflect local cultural characteristics, we comprehensively collected data on dietary intake and factors influencing food consumption, especially household wealth and behavioral lifestyles. Moreover, we adjusted for potential confounders to enhance the reliability of our findings. However, this study has several limitations that should be acknowledged. First, the cross-sectional design precludes the establishment of causal relationships between behavioral lifestyles, dietary patterns and chronic diseases, and only collected single-time-point dietary data, we cannot rule out reverse causation related to dietary changes before and after disease diagnosis. Second, the use of self-reported questionnaire data may introduce information bias. Third, dietary intake, smoking, alcohol consumption, local tea drinking and physical exercise were evaluated qualitatively without precise quantifying their exposure levels, which may restrict the interpretability of the results. Furthermore, the lack of adjustment for total energy intake as a covariate in the multivariable models may have a certain impact on the estimated associations. Finally, the study population was restricted to adults from three villages in Ali, and should be interpreted with caution when extrapolating to other plateau areas or populations with different dietary and cultural backgrounds. Future studies should adopt longitudinal designs with multi-time-point dietary data and conduct external validation of the FFQ to further enhance the accuracy of dietary assessment.

## Conclusion

In summary, our findings demonstrated that the animal-predominant high protein pattern and the three-high pattern were positively associated with chronic diseases in high-altitude areas, whereas the traditional Tibetan pattern was negatively associated with chronic diseases. These association should be interpreted within the framework of socioecological determinants that shape dietary and behavioral habits in this population: the high-altitude geography and Tibetan culture influence food availability and dietary choices to be compatible with local environmental conditions, and ongoing socio-demographic transitions have introduced more animal-based and processed foods, contributing to the emergence of unhealthy dietary patterns. Unhealthy behavioral factors such as smoking, consumption of sweet tea or butter tea, and body weight gain may diminish the protective effects of the traditional Tibetan pattern. Therefore, we recommend quitting smoking, reducing the intake of sweet tea/butter tea, and promoting regular physical exercise, alongside maintaining healthy dietary habits. Additionally, further prospective studies are essential to establish causal relationships between dietary patterns, socioecological determinants and chronic diseases in high-altitude Tibetan populations.

## Supplementary Information

Supplementary Material 1

## Data Availability

The datasets used and/or analyzed during the current study are available from the corresponding author on reasonable request.

## Abbreviations

DP: Dietary patterns
PCA: Principal component analysis
OR: Odds ratio
CI: Confidence interval
SRH: Self-rated health
SD: Standard deviation

## References

[1] Iddir M, Brito A, Dingeo G, Fernandez Del Campo SS, Samouda H, La Frano MR, et al. Strengthening the Immune System and Reducing Inflammation and Oxidative Stress through Diet and Nutrition: Considerations during the COVID-19 Crisis. Nutrients. 2020;12(6):1562. 10.3390/nu12061562. 32471251 PMC7352291

[2] Kopp W. How Western Diet And Lifestyle Drive The Pandemic Of Obesity And Civilization Diseases. Diabetes metabolic syndrome obesity: targets therapy. 2019;12:2221–36. 10.2147/DMSO.S216791. 31695465 PMC6817492

[3] Kang S, Kang M, Lim H. Global and Regional Patterns in Noncommunicable Diseases and Dietary Factors across National Income Levels. Nutrients. 2021;13(10):3595. 34684595 10.3390/nu13103595 PMC8537506

[4] WHO: Noncommunicable diseases. 2023. 10.3390/nu13103595

[5] Noce A, Romani A, Bernini R. Dietary Intake and Chronic Disease Prevention. Nutrients. 2021;13(4). 10.3390/nu13041358. PMC8072965 33921568

[6] Dasa T. Global, regional, and national comparative risk assessment of 84 behaviournvironmental and occupational, and metabolic risks or clusters of risks for 195 countries and territories, 1990–2017: a systematic analysis for the Global Burden of Disease Study 2017. Lancet. 2018; 392(10159):1923–1994. 10.1016/S0140-6736(18)32225-6 PMC6227755 30496105

[7] Ezzati M, Riboli E. Can noncommunicable diseases be prevented? Lessons from studies of populations and individuals. Science. 2012;337(6101):1482–7. 10.1126/science.1227001. 22997325

[8] NCD Countdown. 2030: worldwide trends in non-communicable disease mortality and progress towards Sustainable Development Goal target 3.4. Lancet. 2018; 392(10152):1072–1088. 10.1016/S0140-6736(18)31992-5 30264707

[9] Siddharthan T, Kalyesubula R, Morgan B, Ermer T, Rabin TL, Kayongo A, et al. The rural Uganda non-communicable disease (RUNCD) study: prevalence and risk factors of self-reported NCDs from a cross sectional survey. BMC Public Health. 2021;21(1):2036. 10.1186/s12889-021-12123-7. 34743687 PMC8572568

[10] Zhou M, Wang H, Zeng X, Yin P, Zhu J, Chen W, et al. Mortality, morbidity, and risk factors in China and its provinces, 1990–2017: a systematic analysis for the Global Burden of Disease Study 2017. Lancet. 2019;394(10204):1145–58. 10.1016/S0140-6736(19)30427-1. 31248666 PMC6891889

[11] Li X, Lu J, Hu S, Cheng KK, De Maeseneer J, Meng Q, et al. The primary health-care system in China. Lancet. 2017;390(10112):2584–94. 10.1016/S0140-6736(17)33109-4. 29231837

[12] Qian CX, Zhao Y, Anindya K, Tenneti N, Desloge A, Atun R, et al. Non-communicable disease risk factors and management among internal migrant in China: systematic review and meta-analysis. BMJ global health. 2021;6(9):e003324. 10.1136/bmjgh-2020-003324. 34593512 PMC8487202

[13] Xu Q, Zhou M, Jin D, Zeng X, Qi J, Yin L, et al. Projection of premature mortality from noncommunicable diseases for 2025: a model based study from Hunan Province, China, 1990–2016. Peerj. 2020;8(5):10298. 10.7717/peerj.10298. PMC7646306 33194444

[14] Hu FB, Liu Y, Willett WC. Preventing chronic diseases by promoting healthy diet and lifestyle: public policy implications for China. Obes Rev. 2011;12(7):552–9. 10.1111/j.1467-789X.2011.00863.x. 21366840

[15] Bertin M, Touvier M, Dubuisson C, Dufour A, Havard S, Lafay L, et al. Dietary patterns of French adults: associations with demographic, socio-economic and behavioural factors. J Hum Nutr dietetics. 2016;29(2):241–54. 10.1111/jhn.12315. 25891903

[16] Corsello A, Pugliese D, Gasbarrini A, Armuzzi A. Diet and Nutrients in Gastrointestinal Chronic Diseases. Nutrients. 2020;12(9):2693. 10.3390/nu12092693. 32899273 PMC7551310

[17] Jayedi A, Soltani S, Abdolshahi A, Shab-Bidar S. Healthy and unhealthy dietary patterns and the risk of chronic disease: an umbrella review of meta-analyses of prospective cohort studies. Br J Nutr. 2020;124(11):1133–44. 10.1017/S0007114520002330. 32600500

[18] Neuhouser ML. The importance of healthy dietary patterns in chronic disease prevention. Nutr Res. 2019;70:3–6. 10.1016/j.nutres.2018.06.002. 30077352 PMC6328339

[19] Bauer UE, Briss PA, Goodman RA, Bowman BA. Prevention of chronic disease in the 21st century: elimination of the leading preventable causes of premature death and disability in the USA. Lancet. 2014;384(9937):45–52. 10.1016/S0140-6736(14)60648-6. 24996589

[20] Katz DL, Meller S. Can we say what diet is best for health? Annu Rev Public Health. 2014;35(1):83–103. 10.1146/annurev-publhealth-032013-182351. 24641555

[21] Zhang J, Wang Z, Du W, Huang F, Jiang H, Bai J, et al. Twenty-Five-Year Trends in Dietary Patterns among Chinese Adults from 1991 to 2015. Nutrients. 2021;13(4):1327. 10.3390/nu13041327. 33923855 PMC8072541

[22] Ezzati M, Riboli E. Behavioral and dietary risk factors for noncommunicable diseases. N Engl J Med. 2013;369(10):954–64. 10.1056/NEJMra1203528. 24004122

[23] Dominguez H, Aging, Patterns D. Nutrients. 2022;14(4):889. 10.3390/nu14040889. 35215539 PMC8879056

[24] Zheng J, Guinter MA, Merchant AT, Wirth MD, Zhang J, Stolzenberg-Solomon RZ, et al. Dietary patterns and risk of pancreatic cancer: a systematic review. Nutr Rev. 2017;75(11):883–908. 10.1093/nutrit/nux038. 29025004 PMC5914454

[25] Margerison C, Riddell LJ, McNaughton SA, Nowson CA. Associations between dietary patterns and blood pressure in a sample of Australian adults. Nutr J. 2020;19(1):5. 10.1186/s12937-019-0519-2. 31937324 PMC6961350

[26] Tucker KL. Dietary patterns, approaches, and multicultural perspective. Appl Physiol Nutr Metab. 2010;35(2):211–8. 10.1139/H10-010. 20383235

[27] Samaniego-Vaesken ML, Partearroyo T, Ruiz E, Aranceta-Bartrina J, Gil Á, González-Gross M, et al. The Influence of Place of Residence, Gender and Age Influence on Food Group Choices in the Spanish Population: Findings from the ANIBES Study. Nutrients. 2018;10(4):392. 10.3390/nu10040392. 29565820 PMC5946177

[28] Madlala SS, Hill J, Kunneke E, Faber M. Adult food choices in association with the local retail food environment and food access in resource-poor communities: a scoping review protocol. BMJ open. 2021;11(8):e044904. 10.1136/bmjopen-2020-044904. 34404696 PMC8372818

[29] Milani F, Bottoni M, Giuliani C, Colombo L, Casiraghi MC, Colombo PS, et al. Alpine Diet in Valmalenco (Lombardy, Italy): Nutritional Features of Spontaneous Plants and Traditional Dishes. Nutrients. 2023;15(8):1988. 10.3390/nu15081988. 37111208 PMC10143808

[30] Fanzo J, Miachon L. Harnessing the connectivity of climate change, food systems and diets: Taking action to improve human and planetary health. Anthropocene. 2023;42:100381. 10.1016/j.ancene.2023.100381. 40476929 PMC10084708

[31] Shi YW, Yang XG, Yuan CZ. Eastern coastal Chinese diet associated with reduced obesity and improved cardiometabolic health. Nat Health. 2026;1:416. 10.1038/s44360-026-00079-0.

[32] Zhao WH, Huang ZP, Zhang X, He L, Willett W, Wang JL, et al. Reproducibility and Validity of a Chinese Food Frequency Questionnaire. Biomed Environ Sci. 2010;38. 10.1016/S0895-3988(11)60014-7.

[33] Nyima P, Zhang L, Li Q. Investigation on the prevalence and influencing factors of chronic diseases in the four towns in Ngari, Tibet. Plateau Sci Res. 2021;5(4):92–8. http://dianda.cqvip.com/Qikan/Article/Detail?id=7106229290.

[34] Zhou C, Li M, Liu L, Zhao F, Cong W, Zhang F. Food Consumption and Dietary Patterns of Local Adults Living on the Tibetan Plateau: Results from 14 Countries along the Yarlung Tsangpo River. Nutrients. 2021;13(7):2444. 10.3389/fnut.2021.743896. 34371952 PMC8308694

[35] Xiao Z, Sun X, Zhaxi D, Zhang F, Ji Y, Cheng T, et al. Distinct Nutrient Intake Style in Inhabitants of Ultra-High-Altitude Areas in North of Tibet, China: A Cross-Sectional Study Based on Newly Developed Tibetan Food Frequency Questionnaires. Front Nutr. 2021;8:743896. 10.3389/fnut.2021.743896. 35004798 PMC8733569

[36] Patberg WR, Rasker JJ. Weather effects in rheumatoid arthritis: from controversy to consensus. A review. J Rhuematol. 2004;31(7):1327–34. 10.1016/j.jbspin.2003.09.001. 15229951

[37] 2013 Fifth National Health Service Survey Analysis Report. 2016. http://www.nhc.gov.cn/ewebeditor/uploadfile/2016/10/20161026163512679

[38] Ng R, Sutradhar R, Yao Z, Wodchis WP, Rosella LC. Smoking, drinking, diet and physical activity-modifiable lifestyle risk factors and their associations with age to first chronic disease. Int J Epidemiol. 2020;49:113–30. 10.1093/ije/dyz078. 31329872 PMC7124486

[39] Wang Z, Dang S, Xing Y, Li Q, Yan H. Dietary patterns and their associations with energy, nutrient intake and socioeconomic factors in rural lactating mothers in Tibet. Asia Pac J Clin Nutr. 2017;26(3):450–6. 28429910 10.6133/apjcn.012016.13

[40] Li Y, Nima Q, Yu B, Xiao X, Zeng P, Suolang D, et al. Determinants of self-rated health among an older Tibetan population in a Chinese plateau area: analysis based on the conceptual framework for determinants of health. BMC Public Health. 2021;21(1):489. 10.1186/s12889-021-10359-x. 33706725 PMC7953750

[41] Michishita R, Matsuda T, Kawakami S, Tanaka S, Kiyonaga A, Tanaka H, et al. Long-term Body Weight Gain After Maturity is Associated With the Incidence of Chronic Kidney Disease (CKD), Independent of Current Body Weight, in Middle-aged and Older Men. J Epidemiol. 2019;29(6):213–9. 10.2188/jea.JE20170304. 30344194 PMC6522393

[42] Lavie CJ, Ozemek C, Carbone S, Katzmarzyk PT, Blair SN. Sedentary Behavior, Exercise, and Cardiovascular Health. Circul Res. 2019;124(5):799–815. 10.1161/CIRCRESAHA.118.312669. 30817262

[43] Yu W, Pan L, Cao W, Lv J, Guo Y, Pei P, et al. Dietary Patterns and Risk of Chronic Obstructive Pulmonary Disease among Chinese Adults: An 11-Year Prospective Study. Nutrients. 2022;14(5):996. 10.3390/nu14050996. 35267971 PMC8912729

[44] Huang L, Wang Z, Wang H, Zhao L, Jiang H, Zhang B, et al. Nutrition transition and related health challenges over decades in China. Eur J Clin Nutr. 2021;75(2):247–52. 10.1038/s41430-020-0674-8. 32620907

[45] Chen L, Zhu H, Gutin B, Dong Y, Race, Gender F, Structure S, Status. Dietary Patterns, and Cardiovascular Health in Adolescents. Curr developments Nutr. 2019;3(11):nzz117. 10.1093/cdn/nzz117. PMC6856469 31750413

[46] Quintero-Lesmes DC, Herran OF. Food Changes and Geography: Dietary Transition in Colombia. Annals global health. 2019;85(1):28. 10.5334/aogh.1643. PMC6634597 30873782

[47] Yang Q, Yu S, Wang C, Gu G, Yang Z, Liu H, et al. Health literacy and its socio-demographic risk factors in Hebei: A cross-sectional survey. Medicine. 2021;100(21):e25975. 10.1097/MD.0000000000025975. 34032709 PMC8154485

[48] Arora NK, Pillai R, Dasgupta R, Garg PR. Whole-of-society monitoring framework for sugar, salt, and fat consumption and noncommunicable diseases in India. Ann N Y Acad Sci. 2014;1331:157–73. 10.1111/nyas.12555. 25335459

[49] Ma Y, He FJ, MacGregor GA. High salt intake: independent risk factor for obesity? Hypertension. 2015; 66(4):843–9. 10.1161/HYPERTENSIONAHA.115.05948 26238447

[50] Yau A, Berger N, Law C, Cornelsen L, Greener R, Adams J, et al. Changes in household food and drink purchases following restrictions on the advertisement of high fat, salt, and sugar products across the Transport for London network: A controlled interrupted time series analysis. PLoS Med. 2022;19(2):e1003915. 10.1371/journal.pmed.1003915. 35176022 PMC8853584

[51] Trichopoulou A, Costacou T, Bamia C, Trichopoulos D. Adherence to a Mediterranean diet and survival in a Greek population. N Engl J Med. 2003;348(26):2599–608. 10.1056/NEJMoa025039. 12826634

[52] Jardim MZ, Costa BVL, Pessoa MC, Duarte CK. Ultra-processed foods increase noncommunicable chronic disease risk. Nutr Res. 2021;95:19–34. 10.1016/j.nutres.2021.08.006. 34798466

[53] Kramer H. Diet and Chronic Kidney Disease. Adv Nutr. 2019;10:s367–79. 10.1093/advances/nmz011. 31728497 PMC6855949

[54] Zhang S, Gu Y, Bian S, Górska MJ, Zhang Q, Liu L, et al. Dietary patterns and risk of non-alcoholic fatty liver disease in adults: A prospective cohort study. Clin Nutr. 2021;40(10):5373–82. 10.1016/j.clnu.2021.08.021. 34560608

[55] Shu L, Zheng PF, Zhang XY, Si CJ, Yu XL, Gao W, et al. Association between Dietary Patterns and the Indicators of Obesity among Chinese: A Cross-Sectional Study. Nutrients. 2015;7(9):7995–8009. 10.3390/nu7095376. 26393646 PMC4586571

[56] Zhai H, Wang Y, Jiang W. Fruit and Vegetable Intake and the Risk of Chronic Obstructive Pulmonary Disease: A Dose-Response Meta-Analysis of Observational Studies. BioMed research international. 2020; 2020:3783481. 10.1155/2020/3783481 PMC7057001 32219131

[57] Estruch R, Salas-Salvadó J. Towards an even healthier Mediterranean diet. Nutr metabolism Cardiovasc Dis Nmcd. 2013;23(12):1163–6. 10.1016/j.numecd.2013.09.003. 24263037

[58] Schulze MB, Martínez-González MA, Fung TT, Lichtenstein AH, Forouhi NG. Food based dietary patterns and chronic disease prevention. BMJ. 2018;361:k2396. 10.1136/bmj.k2396. 29898951 PMC5996879

[59] Kahleova H, Levin S, Barnard ND. Vegetarian Dietary Patterns and Cardiovascular Disease. Progress in cardiovascular diseases. 2018; 61(1):54–61. 10.1016/j.pcad.2018.05.002 29800598

[60] Scoditti E, Massaro M, Garbarino S, Toraldo DM. Role of Diet in Chronic Obstructive Pulmonary Disease Prevention and Treatment. Nutrients. 2019;11(6):1357. 10.3390/nu11061357. 31208151 PMC6627281

[61] Slavin JL, Lloyd B. Health benefits of fruits and vegetables. Adv Nutr. 2012;3(4):506–16. 10.3945/an.112.002154. 22797986 PMC3649719

[62] Yang CS, Hong J. Prevention of chronic diseases by tea: possible mechanisms and human relevance. Annu Rev Nutr. 2013;33:161–81. 10.1146/annurev-nutr-071811-150717. 23642203

[63] Prasanth MI, Sivamaruthi BS, Chaiyasut C, Tencomnao T. A Review of the Role of Green Tea (Camellia sinensis) in Antiphotoaging, Stress Resistance, Neuroprotection, and Autophagy. Nutrients. 2019;11(2):474. 10.3390/nu11020474. 30813433 PMC6412948

[64] Wang S, Moustaid-Moussa N, Chen L, Mo H, Shastri A, Su R, et al. Novel insights of dietary polyphenols and obesity. J Nutr Biochem. 2014;25(1):1–18. 10.1016/j.jnutbio.2013.09.001. 24314860 PMC3926750

[65] Türközü D, Şanlier N. L-theanine, unique amino acid of tea, and its metabolism, health effects, and safety. Crit Rev Food Sci Nutr. 2017;57(8):1681–7. 10.1080/10408398.2015.1016141. 26192072

