# Supplementary material for: Socioecological determinants of chronic disease multimorbidity in high-altitude Tibetan populations: a cross-sectional study
Source: J Health Popul Nutr. 2026 Jun 18;45:205. doi: 10.1186/s41043-026-01358-y (PMC13556025; doi:10.1186/s41043-026-01358-y)
Supplement: Supplementary file 1 [file 41043_2026_1358_MOESM1_ESM.docx]

**Supplementary materials**

**Socioecological determinants of chronic disease multimorbidity among high-altitude Tibetan populations: A cross-sectional study on diet-lifestyle-environment interactions**

1. **Supplementary Figures**

**Fig. S1** Flow chart for investigation.

**Fig.S2** The top 10 chronic diseases in Ali area, 2021.

**Fig. S3**. Diseases of sick organ system in Ali area, 2021.

2. **Supplementary Table**

**Table S1** Characteristics of participants in Ali area, 2021 (*n* = 606).

| Variables | Number | Proportion  (%) | Prevalence (n, %) | *P*-value |
| --- | --- | --- | --- | --- |
| Total | 606 | 100.0 | 242 (39.9) |  |
| Gender |  |  |  | 0.466 |
| Male | 329 | 54.3 | 127 (38.6) |  |
| Female | 277 | 45.7 | 115 (41.5) |  |
| Ethnic group |  |  |  | 0.003 |
| Other | 113 | 18.6 | 31 (27.4) |  |
| Tibetan | 493 | 81.4 | 211 (42.8) |  |
| Age |  |  |  | <0.001 |
| ≤ 29 | 175 | 28.9 | 40 (22.9) |  |
| 30-49 | 264 | 43.6 | 98 (37.1) |  |
| ≥ 50 | 167 | 27.5 | 104 (62.3) |  |
| Education level |  |  |  | <0.001 |
| Primary school and below | 273 | 45.1 | 151 (55.3) |  |
| Middle school | 168 | 27.7 | 49 (29.2) |  |
| College and above | 165 | 27.2 | 42 (25.5) |  |
| Marital status |  |  |  | 0.530 |
| In marriage | 422 | 69.6 | 172 (40.8) |  |
| Non-in marriage | 184 | 30.4 | 70 (38.0) |  |
| Employment status |  |  |  | <0.001 |
| Administration and management | 126 | 20.8 | 48 (38.1) |  |
| Professional technicians | 140 | 23.1 | 31 (22.1) |  |
| Housework | 156 | 25.7 | 68 (43.6) |  |
| Other | 184 | 30.4 | 95 (51.6) |  |
| Household wealth |  |  |  | 0.001 |
| Low | 207 | 34.2 | 98 (47.3) |  |
| Medium | 214 | 35.3 | 91 (42.5) |  |
| High | 185 | 30.5 | 53 (28.6) |  |
| Number of family members* |  |  |  | 0.350 |
| 0-3 | 309 | 51.5 | 118 (38.2) |  |
| ≥ 4 | 291 | 48.5 | 122 (41.9) |  |
| Smoking |  |  |  | 0.831 |
| No | 511 | 84.3 | 205 (40.1) |  |
| Yes | 95 | 15.7 | 37 (38.9) |  |
| Alcohol drinking |  |  |  | 0.989 |
| No | 521 | 86.0 | 208 (39.9) |  |
| Yes | 85 | 14.0 | 34 (40.0) |  |
| Tea drinking |  |  |  | 0.005 |
| No | 179 | 29.5 | 56 (31.3) |  |
| Yes | 427 | 70.5 | 186 (43.6) |  |
| Physical exercise |  |  |  | <0.001 |
| No | 393 | 64.9 | 181 (46.1) |  |
| Yes | 213 | 35.1 | 61 (28.6) |  |
| Body weight change in the past year |  |  |  | 0.385 |
| No change | 446 | 73.6 | 172 (38.6) |  |
| Weight gain ≥ 2.5 kg | 86 | 14.2 | 40 (46.5) |  |
| Weight loss ≥ 2.5 kg | 74 | 12.2 | 30 (40.5) |  |
| Self-rated health |  |  |  | <0.001 |
| Well | 354 | 58.4 | 92 (26.0) |  |
| General | 166 | 27.4 | 84 (50.6) |  |
| Poor | 86 | 14.2 | 66 (76.7) |  |

Note: Prevalence was the prevalence of one or more chronic diseases. *six persons were missing in number of family members.
